# Efficient, Fine-Grained Fly Ash Concrete Based on Metal and Basalt Fibers

**DOI:** 10.3390/ma16113969

**Published:** 2023-05-25

**Authors:** Leonid Dvorkin, Janusz Konkol, Vitaliy Marchuk, Andriy Huts

**Affiliations:** 1 Institute of Civil Engineering and Architecture, National University of Water and Environmental Engineering, 33028 Rivne, Ukraine; l.i.dvorkin@nuwm.edu.ua (L.D.); v.v.marchuk@nuwm.edu.ua (V.M.); 2 Faculty of Civil and Environmental Engineering and Architecture, Rzeszow University of Technology, 35959 Rzeszow, Poland; jk7@prz.edu.pl

**Keywords:** Portland cement, fiber concrete, basalt fiber, steel fiber, fly ash, mathematical experiment planning

## Abstract

This article presents the results of a study of the physical and mechanical properties of fine-grained fly ash concrete based on a combined reinforcement with steel and basalt fibers. The main studies were conducted using mathematical planning of experiments, which allowed the experiments to be algorithmized in terms of both the amount of experimental work and statistical requirements. Quantitative dependences characterizing the effect of the content of cement, fly ash binder, steel, and basalt fiber on the compressive strength and tensile splitting strength of fiber-reinforced concrete were obtained. It has been shown that the use of fiber can increase the efficiency factor of dispersed reinforcement (the tensile splitting strength to compressive strength ratio). To increase the resistance of basalt fiber, it is proposed to use fly ash in cement systems, which reduces the amount of free lime in the hydrating cement environment.

## 1. Introduction

An increase in concrete strength leads to increased fragility and deterioration of the deformation properties. This concrete is characterized by almost immediate destruction under short- and long-term loads when it reaches its ultimate stresses [1,2,3,4]. One of the ways to solve this problem is by adding dispersed fiber reinforcement to the cement matrix, which can significantly increase its tensile and flexural strength, crack resistance, resistance to impact and vibration, etc. [5,6,7,8]. Fiber-reinforced concrete has increased abrasion resistance as well as resistance to cracking [9].

Dispersed reinforced concrete, or fiber concrete, is a group of composite materials that include short lengths of various fibers in a cement matrix. Different types of materials, such as steel, glass, synthetic materials, carbon, etc., can be used as fiber [10,11,12,13,14]. Steel fibers, as well as nonmetallic fibers, are usually used to reinforce fine-grained concrete or cement stone. Dispersed reinforcement effectively prevents the development of grain cracks only when the distance between the fibers is not greater than 10–12 mm.

The most popular are Portland cement steel fiber-reinforced concrete. This type of fiber can have a different cross-sectional profile, size, and surface [15,16,17,18]. The amount of fiber added to concrete in volume, according to studies by a few authors, ranges from 0.5 to 2% [19,20]. The addition of steel fiber to concrete increases tensile and flexural strength by up to 200% [4,21,22,23], while the impact on compressive strength is less significant, increasing by 15–20% [24].

Due to its higher crack resistance, steel fiber concrete is characterized by an increase in frost and heat resistance by 1.5 to 2 times. Important indicators of steel fiber concrete are increased resistance to abrasion, impact and dynamic resistance, and shrinkage [25].

Several authors have shown [26,27,28] that the use of steel fiber is effective in reinforced concrete structures to increase their crack resistance, in thin-walled structures, and in structures operating under dynamic loads (airfield pavements, highways, seismic structures, etc.).

At the same time, an important problem with the use of steel fiber in concrete is the possibility of the formation of so-called “hedgehogs”, increased “clumping” in the concrete structure, and uneven distribution of fiber, which requires the use of special devices to evenly add fiber to the concrete mixture.

Although steel fiber is used to improve the mechanical properties of concrete, it is still susceptible to corrosion [29], and its high specific consumption encourages the use of more available methods of concrete reinforcement, in particular the use of nonmetallic fibers. Such a fiber is not subject to corrosion and is therefore suitable for concrete structures in corrosive environments. The effect of various nonmetallic fibers on concrete properties has been described in a number of studies [30,31,32,33,34].

One of the most effective types of mineral fibers for dispersed concrete reinforcement is basalt fiber [35]. It is characterized by high strength under all types of stress conditions and the ability to resist significant deformations in the elastic state. A significant increase in the deformability and strength of cement stone occurs due to the elimination of the influence of stress concentration by basalt fibers in places weakened by structural defects (shells, microcracks). Basalt fiber is chemically inert and does not react with salts or dyes, which is why basalt fiber-reinforced concrete is used in the construction of marine structures and decorative concrete.

The results of many studies show that basalt fiber has a small effect on the compressive strength of concrete, but there is a significant increase in the tensile splitting strength [36]. With a basalt fiber content of 2 kg/m^3^, the increase in compressive and flexural strength at the age of 28 days is 25% and 84% higher compared to concrete without fiber. An appropriate fiber content can optimize the internal microstructure of concrete, but an excessive fiber content will lead to agglomeration, causing a decrease in strength.

The authors [37,38] note a decrease in concrete plasticity caused by the addition of basalt fiber. This was due to the larger surface area, which required more cement paste, which reduced the plasticity of the concrete, and the increased friction between the fibers and the concrete during mixing.

Conflicting results have been reported in the literature on the effect of basalt fiber on the compressive strength of concrete. Li et al. [39] noted that 0.5% of basalt fiber by total concrete volume is optimal and can effectively increase impact strength and provide excellent resistance to freeze–thaw and carbonation of concrete. The addition of basalt fiber can prevent aggregate separation and improve the homogeneity of the concrete mixture [29]. Moreover, the addition of basalt fiber can reduce segregation and shrinkage deformations [40].

However, basalt fiber has a significant disadvantage. In the alkaline environment of hardening Portland cement, the fiber surface is destroyed by the formation of shells [41]. In this regard, when reinforcing concrete with basalt fiber, it is necessary to use additives that bind to CaO; in particular, it is advisable to use fly ash from both an environmental and economic point of view [42,43,44].

An important area of concrete research and development is minimizing the use of cement by partially replacing it with various active mineral additives, including those obtained from production waste. Many production wastes have been sufficiently studied to be used as structural components of binders and concretes, which also helps to solve the environmental problem and accompanies a reduction in the cost of concrete and mortars.

Fly ash is one of the most common mineral fillers, the effectiveness of which has been confirmed by practical experience [45,46]. Thermal power plants fired by crushed hard coal capture accumulate a significant amount of it annually, the quality of which depends on its nature, dispersion, and grain composition. Fly ash actively affects the formation of the structure of composite building materials at all stages of hydration and structure formation of cement systems, that is, the sequential transition from the coagulation structure to the formation of a spatial crystal framework [47].

Fly ash, due to its aluminosilicate phase, has pozzolanic activity and chemically interacts with Ca(OH)_2_, which is released during the hydrolysis of cement clinker minerals. Due to the pozzolanic activity, the addition of fly ash to cement–water systems not only increases the volume of hydrate formation but also accelerates the hydrolysis process and increases the degree of cement hydration, which ultimately has a positive effect on the strength of cement stone [42,47].

Fly ash contributes to the sulfate resistance of cement concrete in the same way as other active mineral additives. This can be explained by the fact that the content of the Ca(OH)_2_ formed in fly ash concrete and mortars as a result of the hydration of C_3_S and C_2_S is lower compared to mixtures without fly ash, since Ca(OH)_2_ together with silicates contained in fly ash form calcium hydrosilicates. Additionally, the amount of ettringite decreases as a result of a decrease in the concentration of Ca(OH)_2_ in hardening concrete [48]. Paper [49] reports a study on improving the protective properties of cement mortars by adding fly ash.

The study of the properties of concrete and concrete mixtures using combined reinforcement is also reported in several works [41,50]. Concrete with combined fiber has better plasticity, flexural tensile strength, and increased crack resistance [51].

The study of the properties of concrete and concrete mixtures using steel and basalt fibers is reported in several papers. This concrete has improved physical and mechanical characteristics. However, there is a significant disadvantage to using basalt fiber in concrete. In the alkaline environment of Portland cement curing, the fiber surface is destroyed, leading to the formation of defects. In this regard, as shown in this study, when reinforcing concrete with basalt fiber, it is necessary to use additives that bind CaO (in particular, fly ash) and consequently reduce the alkalinity of the curing environment. Therefore, to achieve superior physical and mechanical properties of concrete, it is recommended to study fine-grained fly ash concrete based on combined reinforcement with steel and basalt fibers.

## 2. Materials and Methods

**Figure 1:**
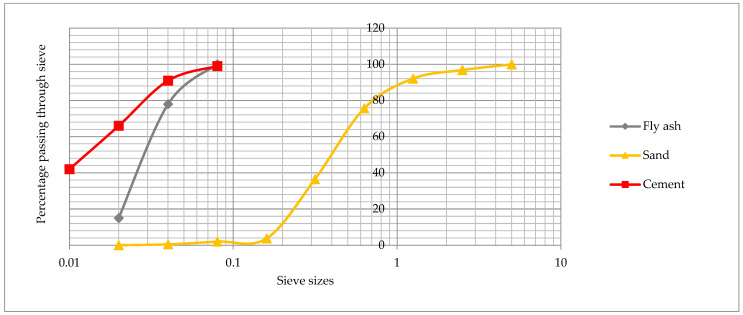
Particle size distribution of cement, fly ash, and sand.

**Figure 2:**
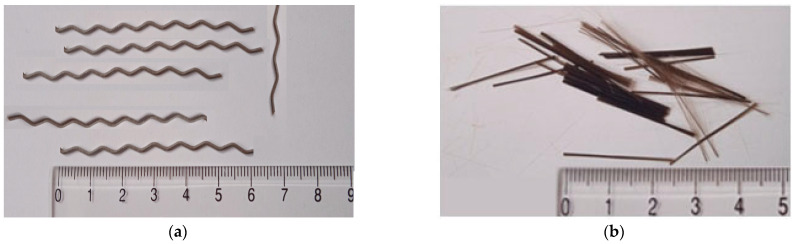
Photo of the fiber used in the research: (**a**) steel fiber; (**b**) basalt fiber.

The materials used for the study of fine-grained fly ash concrete based on combined reinforcement with steel and basalt fibers were:

- Portland cement CEM I 42.5R from the “Dyckerhoff” Wiesbaden, Germany, cement plant in Ukraine. Mineralogical composition of clinker: C_3_S—57.10%; C_2_S—21.27%; C_3_A—6.87%; C_4_AF—12.19% (EN 196-2). The specific surface area of Portland cement S_Cem_ = 300–320 m^2^/kg (EN 196-6). The chemical composition of Portland cement (determined chemically according to EN 196-2) is shown in Table 1; particle size distribution of Portland cement is shown in Figure 1;
- Fly ash from “Burshtyn” TPP (Ivano-Frankivsk Oblast, Ukraine), which is a type II category B ash with a 45 μm sieve particle size of no more than 25% (class 2) (EN 450-1:2012). The specific surface area of the fly ash S_A_ = 250–280 m^2^/kg (EN 196-6). The chemical composition of the fly ash is shown in Table 1; particle size distribution of fly ash is shown in Figure 1;
- Quartz sand with a fineness modulus FM = 2.05. Dust and clay particle content up to 1.5% (EN 12620+A1); particle size distribution of sand is shown in Figure 1;
- Granite aggregate of a 2–5.6 mm fraction. Dust and clay particle content up to 0.5% (EN 12620+A1);
- Polycarboxylate superplasticizer with 30% water reduction effect (EN 934-2);
- Steel fiber (StF) with a wavy shape. The standard tensile strength is 1350 MPa (EN 14889-1). Fiber dimensions: length—60.0 ± 5.0 mm; thickness—1.0 ± 0.1 mm, wave width—6.0 ± 0.1 mm. A photo of the steel fiber is shown in Figure 2a;
- Basalt fiber (BF). Fiber dimensions: length—24 mm, diameter—16 µm. Tensile strength 2800 MPa and elastic modulus 85 GPa. A photo of the basalt fiber is shown in Figure 2b.

In the study of fine-grained fly ash concrete based on combined reinforcement, physical and mechanical research methods, and methods of mathematical planning of experiments were combined. In the course of research to evaluate the effect of fly ash, steel, and basalt fiber on the properties of concrete mixtures and concrete, standard cubes (10 × 10 × 10 cm) and prisms (10 × 10 × 40 cm) were made, which were cured under normal conditions (EN 12390-2). The flowability of the concrete mixture was tested immediately after mixing all components using the fall cone method according to EN 12350-2. The compressive strength was tested on 4 specimens at 7 and 28 days of age (EN 12390-3), and the tensile splitting strength on 3 specimens at 7 and 28 days of age (EN 12390-6). The main studies were conducted using mathematical planning of experiments [52], which allowed the experiments to be algorithmized according to a scheme that is optimal in terms of both the amount of experimental work and statistical requirements. The effect of basalt fiber on the concrete microstructure was studied using an electron microscope of type JSM–5500 LV.

The theory of experiment planning is based on probabilistic and statistical methods that allow for a theoretically grounded determination of the minimum number and composition of experiments, as well as the order of their conduct, to obtain quantitative dependencies between the parameter under study and the factors affecting it.

The task of planning mathematical experiments is to get an idea of the response surface of the factors (graphical dependence), which can be analytically represented as a function:

(1) $$M_{\left\{y\right\}}=\varphi \left(X_{1},X_{2},X_{3},\ldots ,X_{n}\right),\;$$

where *y* is the optimization parameter, that is, the output parameter of the system, and *X_i_* are the variable factors of the same system.

The most convenient way is to represent the unknown function as a polynomial:

(2) $$Y=\beta_{0}+\sum_{i=1}^{n}\beta_{i}X_{i}+\sum_{i=1}^{n}\beta_{ii}X_{i}^{2}+\sum_{i\neq j}^{}\beta_{ij}X_{i}^{}X_{j}^{}+\ldots$$

where *X*_1_, *X*_2_, …, *X_k_* are independent variables (factors) that can be varied in the experiments; *β*_0_, *β_i_*, *β_ij_*, *β_ii_* are theoretical regression coefficients.

The experiment was planned according to a typical matrix, that is, a table with *n* rows and *m* columns, which contains a set of combinations of factors varied relative to some origin or zero (baseline) level. The allowable area of variation of factors (factor space) is selected on the basis of a preliminary study of the object in accordance with the purpose. To simplify the recording of experimental conditions and processing of experimental data, the upper level of factors is coded +1, the lower level −1, and the main level corresponds to 0.

To build quadratic models, a full factorial experiment was used, which provides all possible combinations of factors at three levels. For the technological analysis and selection of significant factors, along with checking the adequacy of the equation, the significance of the regression coefficients was also assessed. The significance of the *b_i_*-regression coefficients was evaluated by finding the experimental value of the *t*-test (*t_i_*) and comparing it with the tabulated value. The regression equations, being quadratic in nature, allow one to trace the individual and joint influence of factors on the studied output parameters to establish the necessary and optimal values of the factors.

The results of the experiments were processed using mathematical statistics methods, obtaining, in general, quadratic regression equations for *k* factors.

## 3. Results and Discussion

In order to study the influence of composition factors on the strength characteristics of fine-grained fly ash fiber concrete as well as establish optimal parameters for the manufacture of such concrete, the main studies were performed using mathematical planning of experiments. For this purpose, a three-level, three-factor plan *B*_3_ was implemented [52], the planning conditions of which are given in Table 2.

The cement–ash binder was obtained by thoroughly mixing Portland cement with fly ash in an amount of 30% by weight of the binder. To reduce the water consumption of the mixture, a polycarboxylate-type superplasticizer was added in an amount of 0.7% by weight of the binder (Portland cement + fly ash). The choice of fiber content was based on the optimal amount of fiber, according to previous studies and a review of the literature [41].

The flowability of the mixtures at all points of the plan was constant and amounted to 12–15 cm, according to the slump of a standard Abrams cone. For this purpose, the amount of water was determined separately for each mixture. The matrix for the planning of experiments and compositions of fly ash concrete mixtures is given in Table 3, and the experimental results of studies of fine-grained fly ash concrete based on combined reinforcement with steel and basalt fibers are given in Table 4.

After processing and statistical analysis of the experimental results obtained, experimental and statistical models of the compressive and tensile strengths of fine-grained fly ash-containing, fiber-reinforced concrete were constructed, which are given in Table 5.

Analysis of constructed models (3–6) confirms that the most significant effect on compressive strength is exerted by the cement–ash binder content, which in turn is associated with the water–binder ratio, and on tensile splitting strength, by dispersed reinforcement. According to the significance of the influence of variable factors on the compressive and tensile splitting strength of concrete, they can be organized in the following descending order: *X*_1_ >> *X*_2_ >> *X*_3_ and *X*_2_ > *X*_3_ > *X*_1_, respectively.

It should be noted that, In order to achieve the required flowability, concrete mixtures require more water to mix with an increase in fiber content. The decrease in concrete plasticity is explained by the larger surface area, which requires more cement paste, while the effect of basalt fiber is more significant.

**Figure 3:**
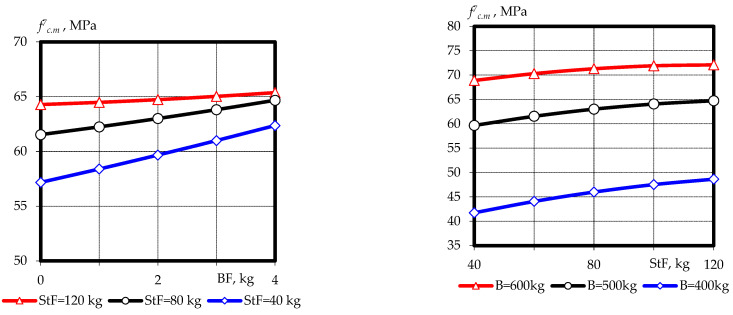
Graphical dependences of the compressive strength of fine-grained fly ash fiber concrete at the age of 7 days.

**Figure 4:**
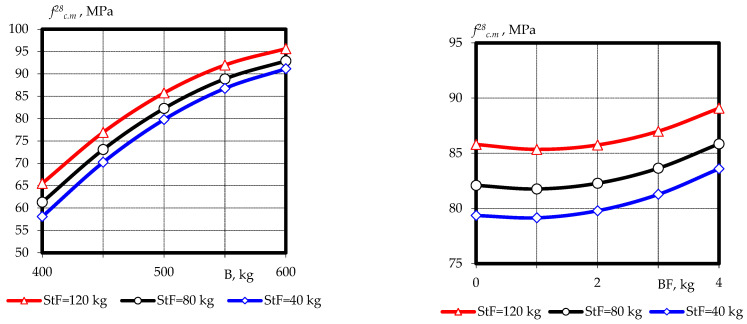
Graphical dependences of the compressive strength of fine-grained fly ash fiber concrete at the age of 28 days.

**Figure 5:**
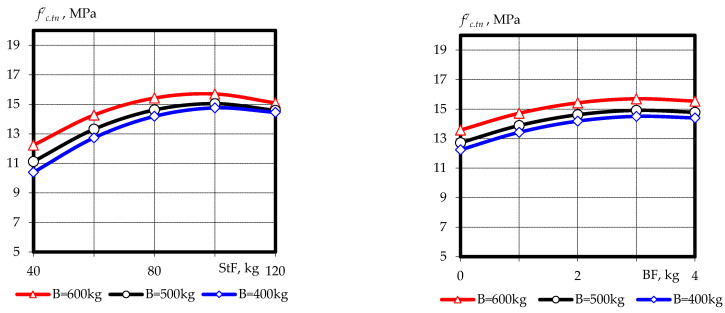
Graphical dependences of the tensile splitting strength of fine-grained fly ash fiber concrete at the age of 7 days.

**Figure 6:**
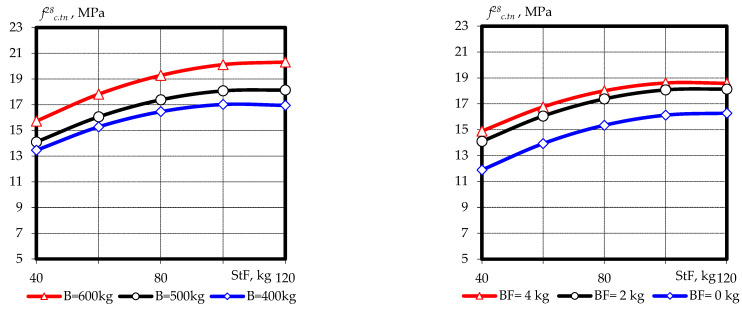
Graphical dependences of the tensile splitting strength of fine-grained fly ash fiber concrete at the age of 28 days.

Based on experimental and statistical models, graphical dependences of compressive strength (Figure 3 and Figure 4) and tensile splitting strength (Figure 5 and Figure 6) of fine-grained fly ash-containing fiber concrete were constructed at the ages of 7 and 28 days, as well as the ratio of tensile splitting strength to compressive strength at the age of 28 days.

The analysis of the results and the graphical dependencies (Figure 3 and Figure 4) allowed us to conclude that the most significant factor affecting the compressive strength of fine-grained fly ash-containing fiber concrete at different curing times is the binder content, the increase in which, from B = 400 kg/m^3^ to B = 600 kg/m^3^, led to an increase in strength of 35–45% at 7 days and 30–40% at 28 days, respectively. This is mainly due to a decrease in the water binding ratio from W/B = 0.38–0.4 to W/B = 0.21–0.24. An increase in steel fiber content within a variable range in a constant water–binder ratio increased compressive strength by 12–16%. The effect was more significant in the absence of basalt fiber and binder volume consumption of up to 500 kg/m^3^. In turn, the effect of basalt fiber at a rate of up to 2 kg/m^3^ did not lead to an increase in compressive strength as occurred at the ages of 7 and 28 days. Further increasing the basalt fiber content to 4 kg/m^3^ yielded a slight increase in compressive strength of up to 10%. As in the case of steel fiber, it is more significant with minimal use of another type of fiber and binder consumption.

The effect of dispersed reinforcement on tensile splitting strength was somewhat different and had a more significant impact (Figure 5 and Figure 6). Increasing the content of steel fiber from 40 kg/m^3^ to 120 kg/m^3^ and basalt fiber to 4 kg/m^3^ led to an increase in tensile splitting strength at 7 days from 8.2–10.5 Mpa to 13.1–15.2 Mpa, depending on the variation of other factors within the experimental plan.

At the same time, the effect on tensile splitting strength at the age of 28 days was somewhat different, whereby, along with the fiber content (both steel and basalt), the cement–ash binder content had a significant impact. The influence of the interaction of factors *X*_1_ with *X*_2_ and *X*_2_ with *X*_3_ was also observed. It should be noted that the positive effect of increasing the fiber content of both types gradually faded as the content approached maximum within the range of variation. This allowed us to assert the so-called optimum zone (the extremes of the graphs), which is in the range of 80 to 100 kg and 2 to 3 kg for steel and basalt fiber, respectively. Under these conditions, the tensile splitting strength reaches 15–17 Mpa at the minimum binder consumption and 17–20 Mpa at the maximum binder consumption.

The results obtained indicate that an increase in fiber content in the entire range of variation leads to an increase in the tensile splitting strength at all curing times and has an extreme character. This indicates a certain optimal consumption of steel fiber (80 to 100 kg) in a composition with basalt fiber (2 to 3 kg), which would ensure the achievement of a maximum tensile splitting strength of 18 to 21 Mpa.

**Figure 7:**
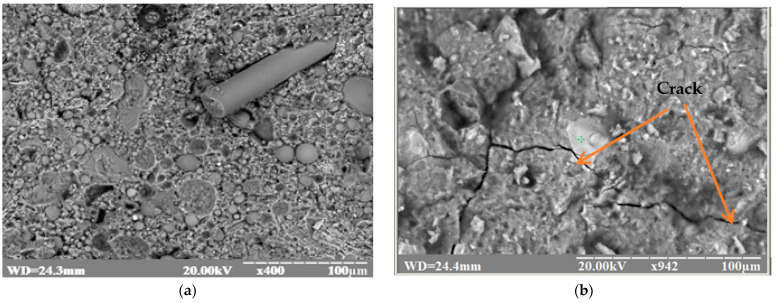

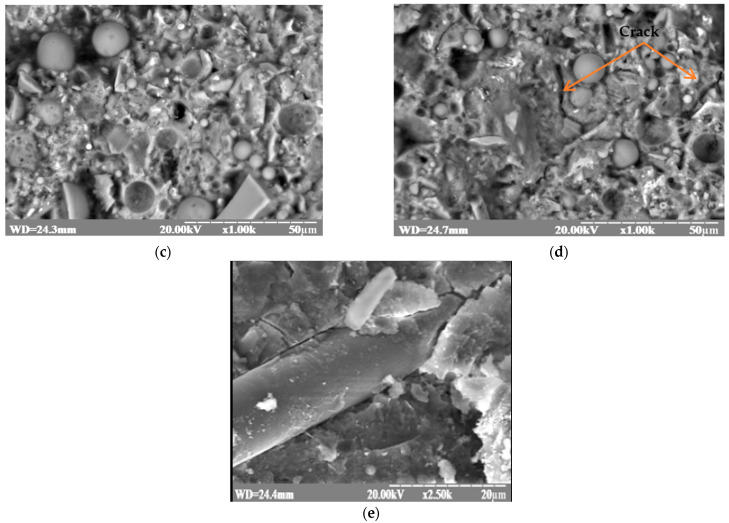
Microstructure of cement–ash stone with the use of composite fiber reinforcement (**a**,**c**,**e**) and without fiber (**b**,**d**).

The increase in strength is explained by the increased resistance to the development of microcracks present in the concrete matrix (Figure 7b,d) due to the presence of fibers.

Thus, as a result of experimental studies, it was established that fine-grained fly ash concrete is possible using steel and basalt fiber characterized by a compressive strength of more than 80 Mpa and a tensile splitting strength of more than 17 Mpa. Combined concrete reinforcement increases the strength of concrete, especially the tensile splitting strength, which can be explained by the improvement of the concrete pore structure.

Given that there is no protective layer on the dispersed reinforcement, the corrosion resistance of fibers in fiber-reinforced concrete is determined mainly by the crack resistance and protective properties of the concrete itself, including permeability, which depend on its pore structure. Improvement in the pore structure and increased crack resistance are more evident when using a finely dispersed basalt fiber.

The micrographs show some of the densely packed hexagonal plates (Figure 7c) that can be attributed to calcium hydroxide. Some spherical ash particles remained in their original state (Figure 7a,c,d), while the bulk of them participated in the hydration process. In the presence of fly ash, which is a source of active Al_2_O_3_ and SiO_2_, crystals of calcium hydrosulfoaluminate 3CaO·AI_2_O_3_·3CaSO_4_·32H_2_O were formed on the surface of sand particles (Figure 7d) and basalt fiber (Figure 7e), which crystallized in the form of needles and provided for the formation of strong bonds between the fly ash particles, cement, and aggregates, increasing the density and mechanical strength of the stone. The high degree of hydration of the binders studied is also evidenced by the nature of pore overgrowth in the cement stone. Hydrate formations in the form of thin crystals of calcium hydrosilicate and elongated crystals of ettringite grew in the pore, contributing to pore colmatization and increasing the strength of the stone. The phase composition of cement stone with fly ash additive is characterized by an increased content of low-base hydrosilicates and calcium hydrosulfoaluminate crystals, which form a homogeneous structure with increased density.

**Figure 8:**
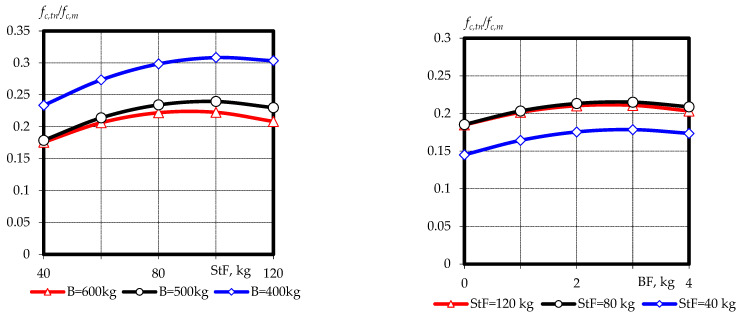
Graphical dependences of the tensile splitting strength to compressive strength ratio of fine-grained fly ash fiber concrete at the age of 28 days.

The deformability of concrete is closely related to its crack resistance. Various criteria have been proposed to characterize the deformability of concrete. One of the simplest is the ratio of tensile splitting, or flexural strength to compressive strength (*f_c.tn_*/*f_c.m_*) [4,41]. This ratio increases as the homogeneity of the concrete micro- and macrostructure increases and the number of various defects contributing to stress concentration decreases. This criterion increases for concrete with dispersed complex reinforcement, which is confirmed by the results obtained in Table 4 and the graphical dependencies in Figure 8. The analysis of the obtained results led us to conclude that for fine-grained fly ash concrete, the increase in *f_c.tn_*/*f_c.m_* from 0.17 to 0.23 and from 0.24 to 0.31 was caused by an increase in the content of fiber, both basalt and steel, at a binder consumption of 400 kg and 600 kg, respectively. However, this tendency was observed at the optimal fiber content. For steel fiber, this is 80–120 kg/m^3^, and for basalt fiber, 2–4 kg/m^3^. This parameter increases significantly with minimum consumption of ash-containing binder in the studied area of variation of the given factor.

It should be noted that unreinforced, fine-grained concrete samples had a brittle fracture pattern. Fiber-reinforced concrete specimens fractured differently, as evidenced by the nature of their destruction. This observation shows that even after crack formation, the fiber-reinforced concrete sample had the ability to carry a certain load. This is due to the fact that some of the fibers did not break at the crack but in the concrete matrix and, after that, were pulled out of the concrete. In addition, there were some fibers anchored on one side of the crack to a small depth. Such fibers were also not cut off but were pulled out of the matrix. The effect described above was enhanced by an increase in the volume content of fiber.

## 4. Conclusions

Based on the analysis of a complex of experimental and statistical models of the strength parameters of efficient fly ash concrete using steel and basalt fiber, the effectiveness of its application for fine-grained concrete has been determined. Quantitative dependences characterizing the effect of the content of the ash-containing binder, steel, and basalt fiber on the tensile splitting strength and compressive strength of fiber-reinforced concrete were obtained.

An increase in fiber content over the entire range of variation leads to an increase in tensile splitting strength at all curing times and has an extreme character, indicating a certain optimal consumption of steel fiber (80–100 kg) in a composition with basalt fiber (2–3 kg), which would ensure the achievement of a maximum tensile splitting strength of 18–21 MPa. The strength at 28 days of the efficient, fine-grained fly ash fiber concrete obtained is within the characteristic limits of high-strength concrete and amounts to 83–98 MPa in compressive strength.

The use of fine-grained concrete on the fiber types studied has been shown to increase the efficiency factor of dispersed reinforcement (the ratio of tensile splitting strength to compressive strength) from 0.15–0.2 to 0.23–0.31.

The replacement of Portland cement with fly ash leads to a slight decrease in compressive and tensile splitting strength, but the addition of composite reinforcement makes it possible to eliminate this deficiency and increase strength properties.

To increase the resistance of basalt fiber in cement systems, it is effective to use fly ash, which reduces the amount of free lime in the hydrating cement environment.

## Figures and Tables

**Table 1:** Chemical composition of Portland cement ^1^ and fly ash.

Material Name	L.O.I.	Oxide Content, %							
		SiO_2_	Al_2_O_3_	Fe_2_O_3_	CaO	MgO	SO_3_	K_2_O	Na_2_O
Clinker	–	21.80	5.32	4.11	66.80	0.95	0.63	0.54	0.42
Fly ash	5.1	46.1	18.1	22.1	2.1	2.0	2.3	1.2	

^1^ The chemical composition of Portland cement based on the clinker used was characterized by an additional SO_3_ content due to the addition of gypsum in the amount of 3.1%.

**Table 2:** Conditions for planning the study experiments.

Technological Factors		Levels of Variation			Variation Interval
Natural View	Coded View	−1	0	+1	
Cement–ash binder content (B), kg/m^3^	*X*_1_	400	500	600	100
Steel fiber content, (StF), kg/m^3^	*X*_2_	40	80	120	40
Basalt fiber content, (BF), kg/m^3^	*X*_3_	0	2	4	2

**Table 3:** Planning matrix and composition of concrete mixtures.

No.	Coded View			Natural View			The Composition of the Concrete Mixture, kg/m^3^					
	*X*_1_	*X*_2_	*X*_3_	Binder	Steel Fiber	Basalt Fiber	Cement ^1^	Fly ash ^1^	Coarse Aggregate	Sand	Water	SP
1	+1	+1	+1	600	120	4	450	150	1030	580	144	4.2
2	+1	+1	−1	600	120	0	450	150	1030	610	132	4.2
3	+1	−1	+1	600	40	4	450	150	1030	680	138	4.2
4	+1	−1	−1	600	40	0	450	150	1030	700	126	4.2
5	−1	+1	+1	400	120	4	300	100	1080	720	160	2.8
6	−1	+1	−1	400	120	0	300	100	1080	730	156	2.8
7	−1	−1	+1	400	40	4	300	100	1080	780	152	2.8
8	−1	−1	−1	400	40	0	300	100	1080	810	144	2.8
9	+1	0	0	600	80	2	450	150	1030	630	138	4.2
10	−1	0	0	400	80	2	300	100	1080	750	160	2.8
11	0	+1	0	500	120	2	375	125	1055	675	135	3.5
12	0	−1	0	500	40	2	375	125	1055	750	125	3.5
13	0	0	+1	500	80	4	375	125	1055	700	135	3.5
14	0	0	−1	500	80	0	375	125	1055	710	125	3.5
15	0	0	0	500	80	2	375	125	1055	710	130	3.5
16	0	0	0	500	80	2	375	125	1055	710	130	3.5
17	0	0	0	500	80	2	375	125	1055	710	130	3.5

^1^ as a part of the binder.

**Table 4:** Experimental results of research.

No.	W/C	Strength, MPa				Crack Resistance ^1^ *f_c,tn_/f_c,m_*
		Tensile Splitting, *f_c,tn_,* at Age		Compressive, *f_c,m_*, at Age		
		7 Days	28 Days	7 Days	28 Days	28 Days
1	0.24	15.3	21.0	73.0	98.4	0.21
2	0.22	13.1	18.8	75.0	96.1	0.20
3	0.23	12.2	16.8	71.0	95.4	0.18
4	0.21	11.1	14.0	75.5	92.1	0.15
5	0.40	13.8	16.8	46.4	68.8	0.24
6	0.39	12.8	15.0	47.5	64.7	0.23
7	0.38	10.9	13.8	45.2	62.8	0.22
8	0.36	8.2	11.2	44.1	57.8	0.19
9	0.23	14.9	17.9	69.0	91.1	0.20
10	0.40	14.7	17.6	47.8	61.1	0.29
11	0.27	15.2	18.2	65.7	86.7	0.21
12	0.25	10.5	13.8	58.2	76.8	0.18
13	0.27	15.4	18.5	64.4	85.0	0.22
14	0.25	12.1	14.6	61.3	80.9	0.18
15	0.26	14.7	17.6	64.0	82.8	0.21
16	0.26	14.4	17.3	62.7	84.5	0.20
17	0.26	14.6	17.5	62.5	82.5	0.21

^1^ tensile strength to compressive strength ratio *f_c,tn_*/*f_cm__._*

**Table 5:** Experimental and statistical models of compressive and tensile splitting strength of fine-grained fly ash fiber concrete.

Parameter	Experimental and Statistical Models	
Compressive strength at the age of 7 days	*f^7^_c,m_* = 63.0 + 12.66∙*X*_1_ + 2.53∙*X*_2_ + 1.57∙*X*_3_ − 0.93∙*X*_1_*X*_2_ − 0.43∙*X*_1_*X*_3_ − 1.03∙*X*_2_*X*_3_ − + 4.35∙*X*_1_^2^ − 0.8∙*X*_2_^2^ + 0.1∙*X*_3_^2^; *R^2^* = 0.974	(3)
Compressive strength at the age of 28 days	*f^28^_c,m_* = 82.28 + 15.79∙*X*_1_ + 2.98∙*X*_2_ + 1.88∙*X*_3_ − 0.73∙*X*_1_*X*_2_ − 0.44∙*X*_1_*X*_3_ − 0.24∙*X*_2_*X*_3_ − + 5.16∙*X*_1_^2^ + 0.49∙*X*_2_*^2^* + 1.69∙*X*_3_^2^; *R^2^* = 0.992	(4)
Tensile splitting strength at the age of 7 days	*f^7^_c__,tn_* = 14.62 + 0.62∙*X*_1_ + 1.73∙*X*_2_ + 1.03∙*X*_3_ − 0.3∙*X*_1_*X*_2_ − 0.05∙*X*_1_*X*_3_ − 0.08∙*X*_2_*X*_3_ + + 0.19∙*X*_1_^2^ − 1.76∙*X*_2_^2^ − 0.86∙*X*_3_^2^; *R^2^* = 0.947	(5)
Tensile splitting strength at the age of 28 days	*f^28^_c__,tn_* = 17.38 + 1.41∙*X*_1_ + 2.02∙*X*_2_ + 1.33∙*X*_3_ + 0.38∙*X*_1_*X*_2_ + 0.08∙*X*_1_*X*_3_ − 0.18∙*X*_2_*X*_3_ + + 0.49∙*X*_1_^2^ − 1.26∙*X*_2_^2^ − 0.71∙*X*_3_^2^; *R^2^* = 0.945	(6)

*R*^2^—coefficient of determination.

## Data Availability

Not applicable.
